# Relationships between cystatin C- and creatinine-based eGFR in Japanese rural community- dwelling older adults with sarcopenia

**DOI:** 10.1007/s10157-020-01981-x

**Published:** 2020-10-22

**Authors:** Hiroshi Kusunoki, Shotaro Tsuji, Tomoyuki Kusukawa, Yosuke Wada, Kayoko Tamaki, Koutatsu Nagai, Masako Itoh, Kyoko Sano, Manabu Amano, Hatsuo Maeda, Hideyuki Sugita, Yoko Hasegawa, Hiromitsu Kishimoto, Soji Shimomura, Ken Shinmura

**Affiliations:** 1 Division of General Medicine, Department of Internal Medicine, Hyogo College of Medicine, 1-1 Mukogawa-cho, Nishinomiya, Hyogo 663-8501 Japan; 2 Department of Orthopaedic Surgery, Hyogo College of Medicine, Nishinomiya, Hyogo Japan; 3 Department of General Medicine and Community Health Science, Sasayama Medical Center Hyogo College of Medicine, Sasayama, Hyogo Japan; 4 Department of Rehabilitation Medicine, Sasayama Medical Center Hyogo College of Medicine, Sasayama, Hyogo Japan; 5 School of Rehabilitation, Hyogo University of Health Sciences, Kobe, Hyogo Japan; 6 School of Pharmacy, Hyogo University of Health Sciences, Kobe, Hyogo Japan; 7 Department of Dentistry and Oral Surgery, Hyogo College of Medicine, Nishinomiya, Hyogo Japan; 8 Division of Comprehensive Prosthodontics, Niigata University Graduate School of Medical and Dental Sciences, Niigata, Niigata Japan

**Keywords:** Sarcopenia, eGFR, AWGS, Cystatin C, Skeletal muscle mass index (SMI)

## Abstract

**Background:**

Sarcopenia is prevalent in patients with chronic kidney disease (CKD). The indices of physical function, such as grip power and gait speed, decreased according to the decline in the estimated glomerular filtration rate (eGFR).

**Methods:**

We examined the relationships between cystatin C-based GFR (eGFRcys), creatinine-based GFR (eGFRcre), their ratio (eGFRcys/eGFRcre) and sarcopenia in community-dwelling older adults in Japan. This cross-sectional study included 302 men aged 73.9 ± 6.2 years and 647 women aged 72.9 ± 5.8 years from a rural area in Hyogo Prefecture, Japan. eGFRcys and eGFRcre were simultaneously measured, and sarcopenia based on the Asia Working Group for Sarcopenia (AWGS) 2019 criteria was evaluated.

**Results:**

eGFRcys and the eGFRcys/eGFRcre ratio were significantly correlated with grip power and gait speed (*p* < 0.001). The eGFRcys/eGFRcre ratio was also correlated with skeletal muscle mass index (SMI) (*p* < 0.01). Univariate logistic regression analysis showed eGFRcys and eGFRcys/eGFRcre ratio but not eGFRcre were associated with sarcopenia (*p* < 0.01). The presence of low eGFRcys (CKDcys) and low eGFRcys/eGFRcre ratio (< 1.0) but not that of low eGFRcre (CKDcre) were associated with sarcopenia (*p* < 0.01). In the multivariate logistic regression analysis, when the eGFRcys/eGFRcre ratio was added as a covariate to the basic model, it was significantly associated with sarcopenia in women (*p* < 0.05). Moreover, low eGFRcys/eGFRcre ratio (< 1.0) was associated with a higher risk of sarcopenia in men (*p* < 0.01).

**Conclusion:**

In conclusion, CKDcys but not CKDcre is associated with sarcopenia. A lower eGFRcys/eGFRcre ratio may be a practical screening marker of sarcopenia in community-dwelling older adults.

## Introduction

Sarcopenia is a disease characterized by loss of skeletal muscle mass (SMM), and it is an important public health problem. Sarcopenia is common in patients with chronic kidney disease (CKD). The muscle strength, for example grip power and physical function such as gait speed, decreased with the decline in the estimated glomerular filtration rate (eGFR) [1]. Serum creatinine (Cr) is a common biomarker that reflects not only renal function, but also systemic muscle mass. Cystatin C (CysC) may be a more reliable biomarker that estimates the glomerular filtration rate (eGFR), and it is not influenced by sex, age, or muscle mass. CKD is defined as reduced eGFR. We previously reported that the Cr/CysC ratio was positively correlated to muscle volume and physical function [2]. Another study has reported that the Cr/CysC ratio was associated with high risk of sarcopenia [3]. A declined eGFR based on CysC (eGFRcys) was associated with a higher prevalence and incidence of frailty, whereas eGFR based on creatinine (eGFRcre) was not [4]. eGFRcys is related to a higher risk of sarcopenia than eGFRcre, because eGFRcys is not influenced by low muscle mass or quality [5].

Kurajoh et al. have reported that a low eGFRcys (CKDcys), but not a low eGFRcre (CKDcre), was independently related to osteoporotic fracture in postmenopausal women. Furthermore, the eGFRcys/eGFRcre ratio was independently related to osteoporotic fracture in this study and was correlated to physical function. The eGFRcys/eGFRcre ratio may be a clinically useful parameter for loss of muscle mass [6]. Several studies have shown the correlation between sarcopenia and osteoporosis [7, 8]. and have suggested a significant correlation between bone and muscle as well as sarcopenia and osteoporosis. Moreover, a prospective study has found that patients with osteoporosis are at an increased risk of developing sarcopenia [9].

We hypothesized that eGFRcys is superior to eGFRcre in evaluating muscle mass and physical function, and is more associated with sarcopenia. The eGFRcys/eGFRcre ratio may provide a clinically relevant measurement of muscle mass. eGFRcre is determined by not only renal function but also muscle mass, thus indicating that a low eGFRcys/eGFRcre ratio is a clinically useful parameter for sarcopenia, because eGFRcre is likely to be overestimated and larger than eGFRcys in sarcopenia [5]. We hypothesized that a eGFRcys/eGFRcre ratio < 1.0 can be related to sarcopenia. Hence, we examined the relationship between eGFRcys and eGFRcre, as well as the relationship between the eGFRcys/eGFRcre ratio and sarcopenia in community-dwelling older adults in Japan.

## Materials and methods

This cross-sectional study was called the Frail Elderly in Sasayama-Tamba Area (FESTA) study. The study population was composed of individuals aged ≥ 65 years. Healthy community-dwelling elderly individuals from the Sasayama-Tamba area, a rural area in Hyogo Prefecture, Japan, were recruited between 2015 and 2019. Physical function assessment, measurement of body composition, and blood sample analysis were performed as described previously [2].

### Categorization of CKD

CKD was defined and classified according to the Kidney Disease: Improving Global Outcomes (KDIGO) criteria [10]. eGFRcre and eGFRcys were calculated using equations by the Japanese Society of Nephrology [11, 12]. Low eGFRcre (CKDcre) is defined as an eGFRcre < 60 mL/min/1.73 m^2^. Low eGFRcys (CKDcys) is defined as an eGFRcys < 60 mL/min/1.73 m^2^.

### Diagnosis of sarcopenia

Sarcopenia was defined according to the criteria for the Asia Working Group for Sarcopenia (AWGS) 2019 [13]. Body composition was evaluated by bioelectrical impedance analysis (BIA) using an InBody 770^®^ (InBody Japan Inc., Tokyo, Japan). The skeletal muscle mass index (SMI) was calculated as SMM/height^2^ (kg/m^2^). The handgrip power, and the normal and maximal gait speed, 5-time chair stand test (5CS), Timed Up and Go test (TUG), and Short Physical Performance Battery (SPPB) scores were evaluated as described previously [2, 13]. Sarcopenia was considered if the participants had a low SMI (< 7.0 kg/m^2^ in men; < 5.7 kg/m^2^ in women) and weak handgrip strength (< 28 kg in men; < 18 kg in women) or low physical performance (normal gait speed < 1.0 m/s, 5CS ≥ 12 s or SPPB ≤ 9).

### Statistical analysis

The results are expressed as mean ± standard deviations (SD) or percentages. For intergroup comparisons, the student’s *t* test was used for data analysis. Pearson's product moment correlation coefficient was used to assess the associations between eGFRcre, eGFRcys and the eGFRcys/eGFRcre ratio and SMM, SMI, body Fat mass (BFM), percentage of BFM, grip power, knee extension muscle strength, normal gait speed, maximal gait speed, and TUG and 5CS scores. Categorical variables were expressed as absolute (*n*) and relative frequency (%) and analyzed by Fisher`s exact test. Univariate and multivariate logistic regression analysis was performed to calculate the odds ratio and 95% confidence interval. A receiver operating characteristic curve (ROC) analysis was performed to confirm the diagnostic efficacy of the eGFRcys/eGFRcre, and the area under the curve (AUC) was calculated. For data analysis, the JMP 13.1 software was used. *p* values < 0.05 were considered significant.

## Results

The baseline characteristics, indices of body composition, and physical performance of the participants are presented in Table 1. The study included 302 men aged 65–94 years, and 647 women aged 65–91 years. The BFM weight and BFM percentages (BFM %) were higher in women than in men. Maximal gait speed, grip power, knee extension muscle strength, SMM, and SMI were higher in men than in women (*p* < 0.001) (Table 1). The average eGFRcre was 69.0 (men: 68.1, and women: 69.3). The average eGFRcys was 74.1 mL/min/1.73 m^2^ (men: 71.7 mL/min/1.73 m^2^, and women: 75.3 mL/min/1.73 m^2^).

**Table 1:** Clinical characteristics, body composition, and physical performance in the subjects

	Total (*n* = 949)	Men (*n* = 302)	Women (*n* = 647)	*p* value
Age (year-old)	73.2 ± 5.9	73.9 ± 6.2	72.9 ± 5.8	0.014
Height (cm)	155.3 ± 8.1	163.9 ± 5.9	151.3 ± 5.6	< 0.001
Body weight (kg)	54.9 ± 9.3	62.6 ± 9.0	51.4 ± 7.0	< 0.001
Body mass index	22.7 ± 2.9	23.3 ± 2.9	22.4 ± 2.8	< 0.001
Skeletal muscle mass (SMM) (kg)	15.7 ± 3.7	19.9 ± 2.7	13.7 ± 1.9	< 0.001
Skeletal muscle mass index (SMI)	6.41 ± 0.93	7.39 ± 0.71	5.96 ± 0.61	< 0.001
Body fat mass (kg)	15.5 ± 5.4	15.0 ± 5.7	15.8 ± 5.2	0.026
Percentage of BFM (%)	28.0 ± 7.6	23.4 ± 6.7	30.2 ± 7.0	< 0.001
Grip power (kg)	27.0 ± 7.3	34.9 ± 6.2	23.4 ± 4.3	< 0.001
Knee extension muscle strength (Nm)	348.7 ± 118.5	450.6 ± 121.1	301.1 ± 81.5	< 0.001
Normal gait speed (m/s)	1.47 ± 0.24	1.45 ± 0.25	1.48 ± 0.24	0.073
Maximal gait speed (m/s)	1.90 ± 0.31	1.96 ± 0.33	1.87 ± 0.30	< 0.001
Timed Up and Go test (TUG)	6.27 ± 1.52	6.25 ± 1.85	6.28 ± 1.34	0.825
5-time chair stand test (5CS)	7.73 ± 4.26	8.21 ± 4.79	7.50 ± 3.99	0.018
Short physical performance battery (SPPB)≦9, *n*(%)	17(1.8)	10(3.3)	7(1.1)	0.795
Cre (mg/dL)	0.72 ± 0.18	0.87 ± 0.12	0.65 ± 0.13	< 0.001
CysC (mg/dL)	0.94 ± 0.20	1.01 ± 0.22	0.90 ± 0.18	< 0.001
eGFRcre (mL/min/1.73 m^2^)	69.0 ± 13.7	68.1 ± 13.5	69.3 ± 13.8	0.205
eGFRcys (mL/min/1.73 m^2^)	74.1 ± 15.6	71.7 ± 15.9	75.3 ± 15.3	0.001
eGFRcys/eGFRcre	1.08 ± 0.17	1.06 ± 0.17	1.10 ± 0.17	0.006
Sarcopenia, *n*(%)	75 (7.9)	25 (8.3)	50 (7.7)	0.797
Hypertension, *n*(%)	418 (44.0)	149 (49.3)	269 (41.6)	0.030
Diabetes, *n*(%)	112 (11.8)	56 (18.5)	56 (8.7)	< 0.001
Dyslipidemia, *n*(%)	213 (22.4)	51 (16.9)	162 (25.0)	0.006
Liver disease, *n*(%)	38 (4.0)	17 (5.6)	21 (3.2)	0.108
Heart disease, *n*(%)	63 (6.6)	31 (10.3)	32 (4.9)	0.003
CKDcre (eGFRcre < 60 mL/min/1.73 m^2^), *n*(%)	225 (23.7)	79 (26.2)	146 (22.6)	0.251
CKDcys (eGFRcys < 60 mL/min/1.73 m^2^), *n*(%)	168 (17.7)	70 (23.2)	98 (15.1)	0.003
eGFRcys/eGFRcre < 1.0, *n*(%)	319 (33.6)	121 (40.1)	198 (30.6)	0.005

Data are expressed as mean ± SD

Among the 949 participants, 75 (25 men and 50 women) participants had sarcopenia based on the AWGS 2019 criteria (Table 1). Other complications (hypertension, diabetes, dyslipidemia, and heart disease) were more prevalent in men than in women (*p* < 0.05). In total, 225 (79 men and 146 women) participants had low eGFRcre (CKDcre), and 168 (70 men and 98 women) had low eGFRcys (CKDcys). In total, 319 (121 men and 198 women) participants had a low eGFRcys/eGFRcre ratio (< 1.0).

The characteristics of all participants are shown in Table 2. Among both men and women, participants with sarcopenia were older than normal participants (*p* < 0.001). Height, body weight, and body mass index (BMI) were lower in participants with sarcopenia than in normal participants, in both men and women (*p* < 0.001). Similarly, normal and maximal gait speed decreased in the participants with sarcopenia (*p* < 0.001). In participants with sarcopenia, muscle strength (grip power and knee extension muscle strength) and muscle volume (SMM and SMI) were lower than in normal participants, in both men and women (*p* < 0.001). TUG and 5CS scores were higher in participants with sarcopenia than in normal participants, irrespective of sex (*p* < 0.01). Body fat mass (BFM) was also lower in participants with sarcopenia than in normal participants, in both men and women (*p* < 0.05), however, there was no significant difference in percentage of BFM between men and women. While there is no difference in Cre value between normal and sarcopenia, CysC value was significantly higher (more than 10%) in sarcopenia subjects in both sexes (*p* < 0.01). eGFRcys but not eGFRcre decreased in both men and women with sarcopenia (*p* < 0.01). The eGFRcys/eGFRcre ratio also decreased in all participants with sarcopenia (*p* < 0.01).

**Table 2:** Characteristics of subjects with and without sarcopenia

	Men			Women		
	Normal (*n* = 277)	Sarcopenia (*n* = 25)	*p* value	Normal (*n* = 597)	Sarcopenia (*n* = 50)	*p* value
Age (year-old)	73.4 ± 5.9	77.7 ± 7.9	0.001	72.5 ± 5.6	77.5 ± 5.9	< 0.001
Height (cm)	164.3 ± 5.8	158.9 ± 5.0	< 0.001	151.8 ± 5.3	145.6 ± 5.4	< 0.001
Body weight (kg)	63.6 ± 8.5	51.9 ± 16.9	< 0.001	51.9 ± 6.9	44.6 ± 5.2	< 0.001
Body mass index	23.5 ± 2.8	20.5 ± 2.5	< 0.001	22.5 ± 2.8	21.0 ± 2.5	< 0.001
Skeletal muscle mass (SMM) (kg)	20.3 ± 2.5	15.9 ± 1.6	< 0.001	13.9 ± 1.8	10.9 ± 1.3	< 0.001
Skeletal muscle mass index (SMI)	7.49 ± 0.64	6.29 ± 0.46	< 0.001	6.02 ± 0.58	5.15 ± 0.36	< 0.001
Body fat mass (kg)	15.2 ± 5.7	12.6 ± 5.2	0.028	16.0 ± 5.2	13.5 ± 4.4	0.011
Percentage of BFM (%)	23.4 ± 5.7	23.6 ± 7.5	0.867	30.2 ± 7.0	29.7 ± 7.0	0.615
Grip power (kg)	35.7 ± 5.7	26.3 ± 4.1	< 0.001	24.0 ± 3.9	16.3 ± 3.1	< 0.001
Knee extension muscle strength (Nm)	462.1 ± 117.6	323.2 ± 80.5	< 0.001	307.5 ± 77.4	224.6 ± 70.1	< 0.001
Normal gait speed (m/s)	1.46 ± 0.24	1.28 ± 0.25	< 0.001	1.49 ± 0.23	1.31 ± 0.34	< 0.001
Maximal gait speed (m/s)	1.98 ± 0.33	1.73 ± 0.27	< 0.001	1.89 ± 0.29	1.62 ± 0.31	< 0.001
Timed Up and Go test (TUG)	6.16 ± 1.79	7.31 ± 2.19	0.003	6.02 ± 0.58	7.41 ± 2.08	< 0.001
5-time chair stand test (5CS)	8.00 ± 4.12	10.85 ± 9.72	0.007	7.23 ± 2.45	10.73 ± 11.20	< 0.001
Short physical performance battery (SPPB)≦9, *n*(%)	4 (1.4)	2 (8.0)	< 0.001	6 (1.0)	5(10.0)	< 0.001
Cre (mg/dL)	0.87 ± 0.17	0.90 ± 0.19	0.374	0.65 ± 0.13	0.66 ± 0.14	0.670
CysC (mg/dL)	1.00 ± 0.21	1.13 ± 0.26	0.003	0.89 ± 0.17	0.97 ± 0.24	0.004
eGFRcre (mL/min/1.73 m^2^)	68.4 ± 13.6	65.0 ± 13.2	0.230	69.5 ± 13.8	67.6 ± 14.1	0.339
eGFRcys (mL/min/1.73 m^2^)	72.5 ± 15.5	62.7 ± 16.9	0.003	75.8 ± 15.1	69.1 ± 17.1	< 0.001
eGFRcys/eGFRcre	1.07 ± 0.17	0.96 ± 0.14	0.002	1.10 ± 0.17	1.02 ± 0.14	< 0.001
CKDcre(+), *n*(%)	70 (25.2)	9 (36.0)	0.242	131 (21.9)	15 (30.0)	0.217
CKDcys(+), *n*(%)	57 (20.6)	13 (52.0)	0.001	83 (13.9)	15 (30.0)	0.006
eGFRcys/eGFRcre < 1.0, *n*(%)	104 (37.5)	17 (68.0)	0.005	177 (29.6)	21 (42.0)	0.079

Data are expressed as mean ± SD

The correlations between eGFRcre, eGFRcys, and the eGFRcys/eGFRcre ratio and the parameters of body composition based on bioelectrical impedance analysis (BIA) (such as SMI, SMM, BFM, and percentage of BFM) and muscle strength and physical function parameters (grip power, knee extension muscle strength, normal gait speed, and maximal gait speed, TUG, 5CS) are shown in Table 3. eGFRcre was not significantly correlated to muscle volume and strength parameters. On the contrary, eGFRcys was positively correlated to SMM and muscle strength parameters in all participants (*p* < 0.05) and was negatively correlated to BFM and percentage of BFM (*p* < 0.001). The eGFRcys/eGFRcre ratio was significantly correlated to SMI, SMM, and muscle strength parameters and exhibited a negative correlation with BFM, percentage of BFM and, TUG and 5CS scores (*p* < 0.05).

**Table 3:** Correlations between eGFRcre, eGFRcys and eGFRcys/eGFRcre and the parameters of body composition based on BIA, muscle strength and physical function parameters

	Men (*n* = 302)						Women (*n* = 647)					
	eGFRcre		eGFRcys		eGFRcys/eGFRcre		eGFRcre		eGFRcys		eGFRcys/eGFRcre	
	r	*p* value	*r*	*p* value	*r*	*p* value	*r*	*p* value	*r*	*p* value	*r*	*p* value
Skeletal muscle mass index (SMI)	− 0.08	0.164	0.13	0.026	0.29	< 0.001	− 0.02	0.631	0.05	0.172	0.12	0.003
Skeletal muscle mass (SMM)	− 0.04	0.446	0.13	0.023	0.26	< 0.001	0.02	0.638	0.13	< 0.001	0.17	< 0.001
Body fat mass	− 0.13	0.024	− 0.25	< 0.001	− 0.18	0.001	− 0.02	0.671	− 0.17	< 0.001	− 0.19	< 0.001
Percentage of BFM	− 0.13	0.023	− 0.29	< 0.001	− 0.25	< 0.001	− 0.02	0.632	− 0.18	< 0.001	− 0.21	< 0.001
Grip power	− 0.06	0.293	0.24	< 0.001	0.43	< 0.001	0.03	0.420	0.21	< 0.001	0.25	< 0.001
Knee extension muscle strength	0.06	0.305	0.28	< 0.001	0.34	< 0.001	0.05	0.206	0.19	< 0.001	0.20	< 0.001
Normal gait speed	0.11	0.067	0.24	< 0.001	0.20	< 0.001	0.10	0.011	0.23	< 0.001	0.19	< 0.001
Maximal gait speed	0.11	0.053	0.29	< 0.001	0.27	< 0.001	0.08	0.040	0.27	< 0.001	0.27	< 0.001
Timed up and go test (TUG)	− 0.16	0.005	− 0.39	< 0.001	− 0.36	< 0.001	− 0.12	0.003	− 0.27	< 0.001	− 0.23	< 0.001
5-time chair stand test (5CS)	− 0.03	0.583	− 0.10	0.078	− 0.11	0.050	0.03	0.445	− 0.06	0.121	− 0.13	< 0.001

**Fig. 1:**
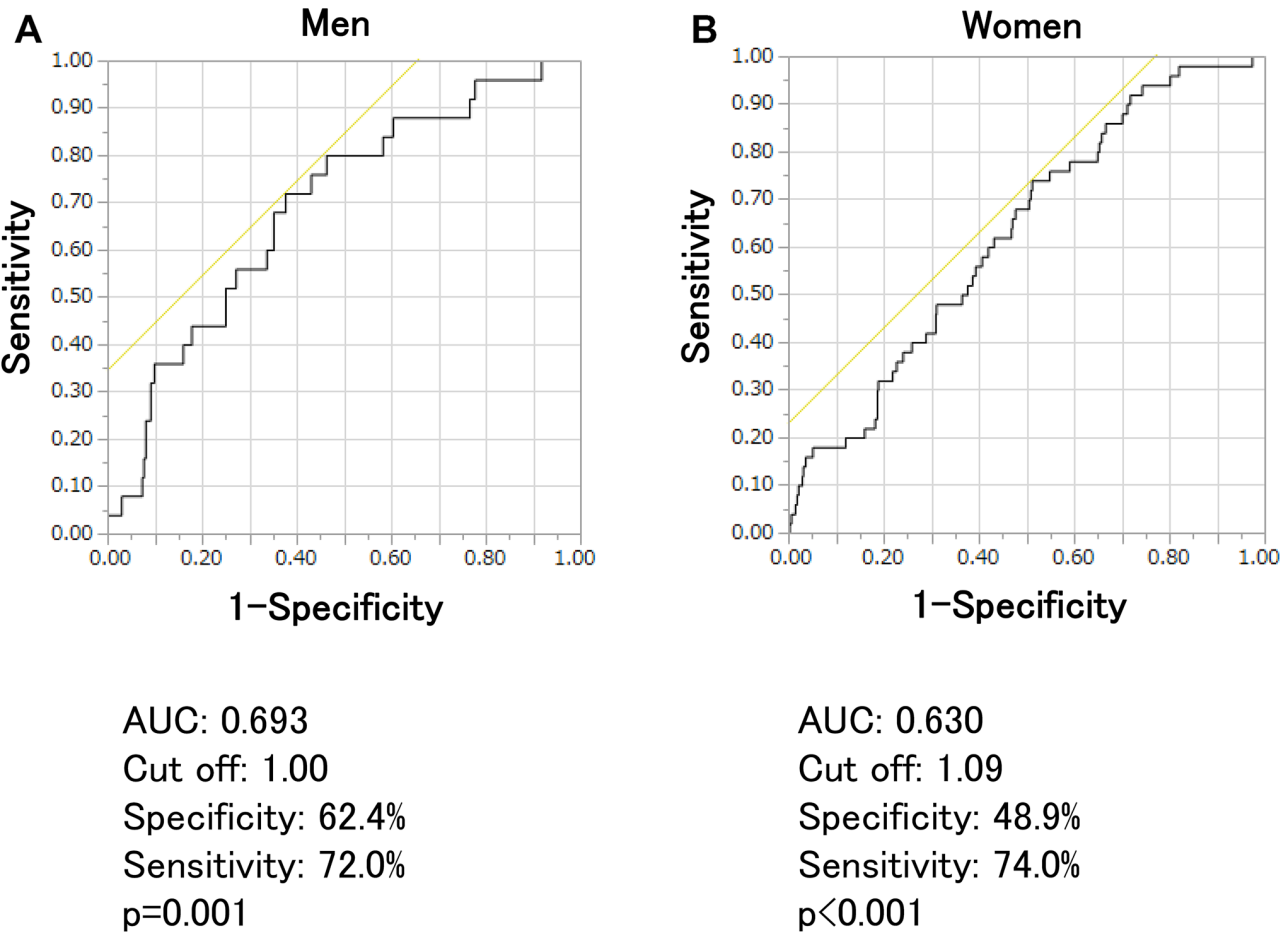
Receiver operating characteristic curves for eGFRcys/eGFRcre ratio and sarcopenia based on the AWGS 2019 criteria

Figure 1 shows the ROC curves of eGFRcys/eGFRcre for identifying sarcopenia in men (A) and women (B). The AUC of eGFRcys/eGFRcre was 0.693 in men and 0.630 in women. The cut-off value was 1.00 in men and 1.09 in women (Fig. 1).

Univariate logistic regression analysis showed eGFRcys and eGFRcys/eGFRcre but not eGFRcre were associated with sarcopenia (*p* < 0.01). Moreover presence of low eGFRcys (CKDcys) and low eGFRcys/eGFRcre ratio (< 1.0) but not that of low eGFRcre (CKDcre) were associated with sarcopenia (*p* < 0.01) (Table 4).

**Table 4:** Univariate logistic regression analysis of factors associated with sarcopenia in men and women

Variables	Men		Women	
	OR (95%CI)	*p* value	OR (95%CI)	*p* value
Age (per 1SD)	1.85 (1.25–2.75)	0.002	2.22 (1.67–2.95)	< 0.001
BMI (per 1SD)	0.25 (0.14–0.44)	< 0.001	0.55 (0.40–0.77)	< 0.001
Hb (per 1SD)	0.72 (0.53–0.96)	0.026	0.91 (0.72–1.14)	0.421
Alb (per 1SD)	0.40 (0.26–0.61)	< 0.001	0.83 (0.61–1.12)	0.226
eGFRcre (per 1SD)	0.77 (0.51–1.18)	0.225	0.87 (0.65–1.16)	0.337
eGFRcys (per 1SD)	0.53 (0.35–0.82)	0.003	0.65 (0.48–0.86)	0.003
eGFRcys/eGFRcre (per 1SD)	0.48 (0.30–0.77)	0.001	0.58 (0.41–0.80)	< 0.001
Hypertension (absence = 0, presence = 1)	0.66 (0.29–1.52)	0.328	1.02 (0.57–1.83)	0.950
Diabetes (absence = 0, presence = 1)	0.58 (0.17–2.00)	0.356	0.91 (0.32–2.63)	0.862
Dyslipidemia (absence = 0, presence = 1)	0.40 (0.09–1.77)	0.179	0.31 (0.12–0.80)	0.005
Liver disease (absence = 0, presence = 1)	1.52 (0.327–7.05)	0.610	1.27 (0.29–5.60)	0.762
Heart disease (absence = 0, presence = 1)	2.41 (0.84–6.97)	0.128	2.35 (0.86–6.38)	0.125
CKDcre (absence = 0, presence = 1)	1.66 (0.70–3.93)	0.257	1.52 (0.81–2.88)	0.204
CKDcys (absence = 0, presence = 1)	4.18 (1.81–9.66)	< 0.001	2.65 (1.39–5.07)	0.005
eGFRcys/eGFRcre < 1.0 (absence = 0, presence = 1)	3.53 (1.47–8.48)	0.003	1.72 (0.95–3.09)	0.076

Multivariate logistic regression analysis was performed to examine whether eGFRcys, eGFRcre, and the eGFRcys/eGFRcre ratio were independently associated with sarcopenia. Although eGFRcys was not significantly associated with sarcopenia, when the eGFRcys/eGFRcre ratio was added as a covariate to the basic model, a significant association was observed between the eGFRcys/eGFRcre ratio and sarcopenia in women (*p* < 0.05) (Table 5).

**Table 5:** Multivariate logistic regression analysis of factors associated with sarcopenia in men (A), in women (B)

	Model 1		Model 2		Model 3		Model 4	
Variables	OR (95%CI)	*p* value	OR (95%CI)	*p* value	OR (95%CI)	*p* value	OR (95%CI)	*p* value
(A) Men								
Age (per 1SD)	1.38 (0.88–2.16)	0.154	1.31 (0.83–2.08)	0.243	1.11 (0.67–1.84)	0.674	1.15 (0.70–1.89)	0.590
BMI (per 1SD)	0.29 (0.16–0.53)	< 0.001	0.26 (0.14–0.50)	< 0.001	0.27 (0.15–0.51)	< 0.001	0.32 (0.18–0.58)	< 0.001
Hb (per 1SD)	1.16 (0.73–1.85)	0.517	1.21 (0.76–1.94)	0.400	1.19 (0.76–1.86)	0.434	1.11 (0.71–1.73)	0.652
Alb (per 1SD)	0.46 (0.28–0.78)	0.003	0.48 (0.29–0.82)	0.005	0.50 (0.29–0.85)	0.010	0.46 (0.27–0.79)	0.004
eGFRcre (per 1SD)			0.74 (0.44–1.26)	0.263				
eGFRcys (per 1SD)					0.60 (0.35–1.05)	0.069		
eGFRcys/eGFRcre (per 1SD)							0.62 (0.34–1.17)	0.135
(B) Women								
Age (per 1SD)	2.55 (1.86–3.51)	< 0.001	2.71 (1.93–3.81)	< 0.001	2.42 (1.68–3.47)	< 0.001	2.31 (1.67–3.20)	< 0.001
BMI (per 1SD)	0.46 (0.32–0.66)	< 0.001	0.46 (0.32–0.67)	< 0.001	0.45 (0.31–0.65)	< 0.001	0.44 (0.30–0.63)	< 0.001
Hb (per 1SD)	1.17 (0.85–1.61)	0.320	1.16 (0.84–1.60)	0.363	1.18 (0.86–1.62)	0.297	1.17 (0.85–1.62)	0.332
Alb (per 1SD)	0.98 (0.69–1.39)	0.901	0.99 (0.70–1.40)	0.939	0.98 (0.69–1.40)	0.938	1.04 (0.73–1.48)	0.834
eGFRcre (per 1SD)			1.18 (0.84–1.67)	0.332				
eGFRcys (per 1SD)					0.89 (0.62–1.29)	0.540		
eGFRcys/eGFRcre (per 1SD)							0.64 (0.44–0.93)	0.014

Model 1 included age, BMI, hemoglobin and albumin as covariates. In other models, eGFRcre (model 2), eGFRcys (model 3) and eGFRcys/eGFRcre ratio (model 4) were added to model 1

Multivariate logistic regression analysis was performed adjusted for complications (hypertension, diabetes, dyslipidemia, liver disease, and heart disease) to examine whether CKDcys and CKDcre were independently associated with sarcopenia. CKDcys was independently associated with sarcopenia both in men and women and an eGFRcys/eGFRcre < 1.0 was independently associated with sarcopenia in men (*p* < 0.01) (Table 6).

**Table 6:** Multivariate logistic regression analysis of CKDcre, CKDcys and eGFRcys/eGFRcre < 1.0 adjusted for complications (hypertension, diabetes, dyslipidemia, liver disease, and heart disease), associated with sarcopenia in men (A), in women (B)

Variables	OR (95%CI)	*p* value
(A) Men		
CKDcre	1.63 (0.67–3.93)	0.287
CKDcys	4.88 (2.00–11.95)	< 0.001
eGFRcys/eGFRcre < 1.0	3.70 (1.52–9.00)	0.003
(B) Women		
CKDcre	1.68 (0.88–3.22)	0.129
CKDcys	2.67 (1.37–5.19)	0.006
eGFRcys/eGFRcre < 1.0	1.50 (0.82–2.74)	0.196

## Discussion

The present study showed that, low eGFRcys (CKDcys) was more frequent in participants with sarcopenia than in normal participants (Table 2). However there was no difference in the frequency of low eGFRcre (CKDcre) between sarcopenia and normal participants. In the multivariate logistic regression analysis adjusted for complications (hypertension, diabetes, dyslipidemia, liver disease, and heart disease), CKDcys was clearly related to sarcopenia based on AWGS 2019 while CKDcre was not (Table 6). CKD is considered a risk factor for several aging-related diseases, including cardiovascular diseases (CVDs), metabolic syndrome, frailty, and sarcopenia. Previous studies have reported that CKDcys was more associated with life prognosis and physical function than CKDcre. In octogenarians, CKDcys was associated with increased odds of CVDs [14]. The Cardiovascular Health Study has shown that elderly individuals with CKDcys had a high risk of heart failure and mortality [15]. In the Framingham Offspring Study, participants with CKDcys had greater gait speed declines than those with CKDcre. The participants with CKDcys also had greater odds of mobility disability than those with CKDcre [16]. These previous reports support our result that, CKDcys is more associated with sarcopenia than CKDcre.

Participants with high muscle mass are likely to have high creatinine levels and low eGFRcre, and participants with sarcopenia are likely to have low creatinine levels and high eGFRcre. In the elderly sarcopenia patients, renal function is overestimated by using eGFRcre. eGFRcys reflects the renal function more accurately than eGFRcre. The relationship between sarcopenia and renal function is established by estimating renal function using eGFRcys.

Second, this study showed the eGFRcys/eGFRcre ratio was significantly positively correlated to SMI, SMM, and muscle strength and physical funtion parameters and was negatively correlated to BFM and percentage of BFM. (Table 3). The eGFRcys/eGFRcre ratio may provide a clinically relevant measurement of muscle mass, based on the assumption that eGFRcre is determined using not only renal function but also muscle mass [5]. To the best of our knowledge, this study is the first study which shows that the eGFRcys/eGFRcre ratio may be a clinically useful parameter for reduced muscle mass in community-dwelling elderly individuals.

The correlation coefficients between eGFRcys/eGFRcre and muscle volume and strength parameters were quite low in this study. In our previous study, the correlation coefficients between Cr/CysC and said parameters were observed to be quite low. For example, the correlation coefficients (r) between Cr/CysC and SMI were *r* = 0.34 (*p* < 0.0001) in men and *r* = 0.08 (*p* = 0.0767) in women [2]. Other studies reported the correlation coefficients between Cr/CysC and muscle volume, they were also not so high. Barreto et al. reported, Cr/CysC correlated with muscle volume evaluated with an abdominal CT scan. The correlation coefficients (*r*) between Cr/CysC and muscle volume was also quite low (*r* = 0.40) [17]. Kashani K et al. reported Cr/CysC correlated with muscle volume in lung transplant candidates, and the correlation coefficients (*r*) were also the same level [18].

Although there are many reports Cr/CysC is associated with muscle volume loss [17–23], we can not estimate the muscle volume or muscle power and diagnosis sarcopenia by only Cr/CysC. This study showed, not only Cr/CysC but also eGFRcys/eGFRcre surely related to muscle volume and muscle power parameters. Because the eGFRcys and eGFRcre measurements are routinely carried out in the clinical setting of managing patients with CKD, our results strongly suggest that there is a possibility that eGFRcys/eGFRcre is useful as a screening tool of sarcopenia. Further investigations are needed for more sophisticated screening tools by using these parameters.

Third, this study showed that in participants with sarcopenia, an eGFRcys/eGFRcre ratio < 1.0 is more frequent than in normal participants (Table 2). In the multivariate logistic regression analysis adjusted for complications, an eGFRcys/eGFRcre ratio < 1.0 was clearly shown to be associated with sarcopenia in men (Table 6).

In the ROC analysis, the cut-off value of the eGFRcys/eGFRcre ratio was 1.00 in men and 1.09 in women. Although the 1.0 cut-off value of the eGFRcys/eGFRcre ratio is easy to evaluate, it may be too low for women. This discrepancy of the cut-off value between men and women may explain the reason why the eGFRcys/eGFRcre ratio < 1.0 found only in men was clearly shown to be associated with sarcopenia (Fig. 1).

The AUC of eGFRcys/eGFRcre was higher in men (0.693) than in women (0.630). Similarly, in a previous study, the AUC of Cr/CysC for identifying sarcopenia was higher in men than in women [23]. Generally, total muscle volume is higher in men than in women. The influence of the change in muscle volume is less in CysC than in Cre. Therefore, the change in eGFRcys/eGFRcre by the decrease in skeletal muscle mass is expected to be bigger in men than in women. The difference in body composition between sexes and the discrepancy in the influence on eGFRcys/eGFRcre by the change in muscle volume between men and women may explain said difference.

There are no participants with CKD stage 5 in both sexes. This result indicates that even in the participants without end-stage renal failure, only “eGFRcre is more than eGFRcys” may be related to sarcopenia. Recently it has been reported that difference between eGFRcre and eGFRcys (dGFR) correlates with muscle strength positively in patients with liver damage [24]. The eGFRcre may become detached from eGFRcys in patients with sarcopenia.

The release of creatine from the muscle is the major determinant of serum Cr levels, due to its conversion to Cr in the circulation. CysC is a cysteine protease inhibitor that is constantly produced by all nucleated cells. Thus, it is unaffected by muscles mass [5] and the eGFRcys value has a lower level of bias. As mentioned in a previous study, CysC may be influenced by mild chronic inflammation and oxidative stress [25, 26]. Thus, eGFRcys may be more sensitive to mild inflammatory and oxidative changes in sarcopenia than eGFRcre. Recently, a significant relationship was observed between the risk for CVD and sarcopenia, because of the increased circulating markers of oxidative stress in sarcopenia [27]. These mechanisms may support our present study. In Japan, CysC levels are widely monitored in daily clinical settings. Moreover, in numerous institutions, both eGFRcre and eGFRcys are calculated automatically by the center clinical inspection section. An eGFRcys/eGFRcre ratio < 1.0 can be evaluated easily in many institutions.

This study has some limitations that must be considered. First, this is a cross-sectional study. Therefore, any cause-and-effect relationships cannot be evaluated. A prospective study must be conducted to assess any causal associations between CKD and sarcopenia. Second, most of the participants in this study voluntarily participated in our study. Thus, the study participants may be healthier, and the study population might have lower rates of sarcopenia than those in the general population. This could account for any inconsistency between our results and previous studies. Third, we did not measure urinary protein. Thus, the association between CKD and sarcopenia that was modified by the presence of subclinical kidney disease was not examined. Finally, a small number of participants with sarcopenia were included in the study, which obviously limits the reliability and applicability of the proposed test.

In conclusion, CKDcys but not CKDcre is associated with sarcopenia. A low eGFRcys/eGFRcre ratio may be a practical screening marker for sarcopenia based on the AWGS 2019 criteria in rural community-dwelling older adults. Further studies are needed to evaluate the diagnostic value of the eGFRcys/eGFRcre ratio for estimating sarcopenia. The prognostic value of the eGFRcys/eGFRcre ratio for predicting clinical outcomes in older adults also warrants further study.

## Abbreviations

CKD: Chronic kidney disease
eGFR: Estimated glomerular filtration rate
Cr: Creatinine
CysC: Cystatin C
BMI: Body mass index
OR: Odds ratio
CI: Confidence interval
SD: Standard deviations
FESTA: Frail Elderly in Sasayama-Tamba Area
KDIGO: Kidney Disease: Improving Global Outcomes
AWGS: Asian Working Group for Sarcopenia
BIA: Bioelectrical impedance analysis
SMM: Skeletal muscle mass
SMI: Skeletal muscle mass index
BFM: Body fat mass
CVDs: Cardiovascular diseases
Hb: Hemoglobin
Alb: Albumin
ROC: Receiver operating characteristic curve
AUC: Area under the curve
